# Role of C reactive protein in evaluating the extent of myocardial inflammation in acute myocarditis

**DOI:** 10.1186/1532-429X-17-S1-P291

**Published:** 2015-02-03

**Authors:** Orly Goitein, Avi Sabag, Rafi Koperstein, Ashraf Hamdan, Elio Di Segni, Eli Konen, Shlomi Matetzky

**Affiliations:** Diagnostic Imaging, Sheba Medical Center, Tel Hashomer, Israel; Heart Center, Sheba Medical Center, Tel Hashomer, Israel

## Background

Cardiac MRI (CMR) is an excellent modality for myocardial tissue characterization, allowing demonstration and quantification of myocardial damage using late Gadolinium enhancement (LGE) sequence. C Reactive Protein (CRP) has been shown to be a prognostic marker in lymphocytic myocarditis. The purpose of this study was to study the correlation between CRP levels and the extent of myocardial damage in patients diagnosed with myocarditis.

## Methods

The study cohort included 47 patients (Mean age 32.5±12, 89% males) admitted to the heart center with MRI proven acute myocarditis. Demographic, clinical, laboratory echocardiographic and CMR data was collected. CMR was performed using a 1.5 T scanner (Signa HDX GE medical systems) using a standardized protocol including SSFP and LGE sequnces. LGE was quantified as percentage of the left ventricular (LV) mass. Patients were divided into two groups: LGE< 10% of LV mass and LGE> 10.1% of LV mass. These two groups were compared in regards to CRP levels and left ventricular (LV) function.

## Results

CRP levels were positively correlated with the myocardial damage; in the LGE< 10% group CRP was 54.6±56, in the and LGE> 10.1% group CRP was 104±68 (P value= 0.022). This correlation was maintained also when CRP was divided into thirtailes (P value=0.004). LV ejection fraction was 59.9%±6.7 VS 52.2%±12 for the lower and mid LGE thirtailes and upper LGE thirtaile, respectively (P value=0.023)

No correlation was found between the extent of myocardial damage (% LGE) and the CPK and Troponin levels or any of the baseline characteristics (table 1).

**Table 1:** Late Gd enhancement (LGE) percentage baseline characteristics and CRP CPK and Troponin levels

	LGE<10% N= 16	LGE≥10% N= 31	P value
Age (Mean ± SD)	30.7±11	34.4±14	0.352
Diabetes mellitus	0	6.5	0.541
Hypertension	0	9.7	0.541
Dyslipidemia	0	16.1	0.150
Obesity	56.3	53.3	1
Smoker	31.3	29	1
CPK	560±680	612±427	0.779
Troponin	19.5±27	12±9.5	0.310
Troponin upper thirtile	31.3	29	1
LVEF			
CRP	54.6±56	104±68	0.022
CRP upper thirtaile	21.4	63	0.012

CPK- Creatinin Phosphosokinase

LVEF- Left Ventricular Ejection Fraction

CRP-C Reactive protein

## Conclusions

CRP was significantly and positively correlated with the extent of myocardial damage as quantified by CMR. It may serve as a simple yet valuable tool to predict LV damage and dysfunction in patients presenting with acute myocarditis.

## Funding

No external funding was used for this study.

